# Editorial: *In vitro* diagnosis of allergic and mast cell-mediated disorders

**DOI:** 10.3389/falgy.2024.1483398

**Published:** 2024-10-25

**Authors:** Joana Vitte, Alexandra F. Santos

**Affiliations:** ^1^ Immunology Laboratory, Biology and Pathology Department, University Hospital of Reims, Reims, France; ^2^ University of Reims Champagne Ardenne, INSERM UMR 1250, Reims, France; ^3^ Department of Women and Children’s Health (Pediatric Allergy), Faculty of Life Sciences and Medicine, School of Life Course Sciences, King’s College London, London, United Kingdom; ^4^ Peter Gorer Department of Immunobiology, School of Immunology and Microbial Sciences, King’s College London, London, United Kingdom; ^5^ Children’s Allergy Service, Evelina London Children’s Hospital, Guy’s and St Thomas’ Hospital, London, United Kingdom

**Keywords:** immunoglobulin (Ig) E, *in vitro* diagnosis of allergy, basophil activation test (BAT), mast cell (MC), hereditary alpha-tryptasemia (HαT), molecular allergen, component-resolved diagnosis (CRD), COVID-19 vaccine

**Editorial on the Research Topic**
*In vitro* diagnosis of allergic and mast cell-related disorders

Allergic diseases, affecting one in four people, are diagnosed through the demonstration of an adaptive immune response (sensitization) to a trigger (allergen) of a hypersensitivity reaction. In clinical practice, most hypersensitivity reactions referred to allergists are immediate reactions, taking place less than 2 h following exposure to the trigger (1). In most cases, immediate hypersensitivity symptoms develop within minutes after ingestion, inhalation, or injection of the trigger. The occurrence and severity of immediate hypersensitivity reactions are influenced by mast cell conditions, such as hereditary alpha-tryptasemia (H*α*T), estimated to affect 5 to 8% of general population (2).

Allergen-induced immune responses can be demonstrated using *in vivo* (skin prick test and provocation tests), and *in vitro* tests. Conventional diagnostic strategies require a two-step diagnostic process: first, taking a detailed clinical history aimed at identifying one or a limited number of suspected triggers; and second, proceeding to complementary tests to confirm sensitization to the suspected trigger(s). This decades-old diagnostic paradigm is increasingly shifting towards a precision medicine approach comprising phenotype stratification, personalized therapeutic decision making, risk prediction and even family counselling (3, 4). The allergic patient is increasingly placed at the center of an integrated approach thanks to progress in endotyping the mechanisms at play in the build-up of the allergic host–environment interaction. Key contributors are modern *in vitro* diagnosis concepts and tools, therapeutic breakthroughs and improved knowledge of environmental factors.

Current *in vitro* diagnostic methods are mostly quantitative and increasingly standardized, allowing for reliable comparison and follow-up of allergic patients (5). They are also miniaturized, with hundreds or even thousands of biomarkers assayed in minute volumes of biological fluids. Non- or minimally- invasive tests are being developed, allowing for better pediatric assessment and lower health care resource utilization. Moreover, new regulatory standards result in improved quality of *in vitro* tests (6).

The Research Topic “*In vitro* diagnosis of allergic and mast cell-mediated disorders” aimed to provide an overview of currently available *in vitro* diagnostic tests for allergic and mast cell-related disorders, and describe their contribution to the prediction, diagnosis and management of patients with a suspected or confirmed allergic or mast cell disorder. Measurement of allergen-specific immunoglobulin (Ig) E, mainly focused on quantitative aspects including the clinical decision associated with low levels of sensitization, and molecular allergen-specific IgE are important tools for phenotype assessment and risk stratification of allergic patients (Balsells-Vives et al., Chantran et al.). Basophil activation test (BAT) provides functional evaluation of a patient's response to IgE-dependent as well as IgE-independent immediate hypersensitivity triggers (reviewed in (Sonder et al.). Using BAT to molecular allergens may improve the diagnostic specificity even further, e.g., discrimination between allergic broncho-pulmonary aspergillosis and *Aspergillus fumigatus* sensitization (Michel et al.). BAT shows promise for the identification of more severe allergic phenotypes at risk of anaphylaxis, such as wheat-dependent exercise-induced anaphylaxis or soy-induced anaphylaxis (Gabler et al., Evrard et al.). However, access to BAT is still limited due to sampling and technical requirements (fresh whole blood samples, flow cytometry facilities, available soluble allergens) and, ideally, need for regulatory approval (Alpan et al.). Serum baseline tryptase determination is, currently, the best screening option for identifying people with H*α*T, a disease-modifying mast cell condition associated with a higher risk of severe immediate hypersensitivity reactions of various causes, including food allergy (Chantran et al.).

An additional aim of this Research Topic was to provide the reader with the pathophysiological and methodological explanation of *in vitro* diagnostic tools, enabling better understanding of future developments in the field for improved diagnosis and management. Here, Stoffersen et al. investigated the relationship between free serum IgE levels and the performance of a modified BAT using patient's serum and control donor basophils (Stoffersen et al.), while Nicaise-Roland et al. reviewed the causes of hypersensitivity reactions to COVID-19 vaccines and the adequate strategies of *in vitro* diagnosis (Nicaise-Roland et al.).

Taken together, the contributions to the Research Topic “*In vitro* diagnosis of allergic and mast cell-mediated disorders” showcase current hotspots, unmet needs and ongoing research in the field of *in vitro* diagnosis of allergic and mast cell disorders. The manuscripts compiled herein illustrate, through selected examples, how the precision medicine approach using up-to-date *in vitro* diagnostic tools is beneficial to allergic patients, with respect to their culprit allergens as well as to their genetic make-up, paving the way of personalized medical and lifestyle interventions.

**Figure 1:**
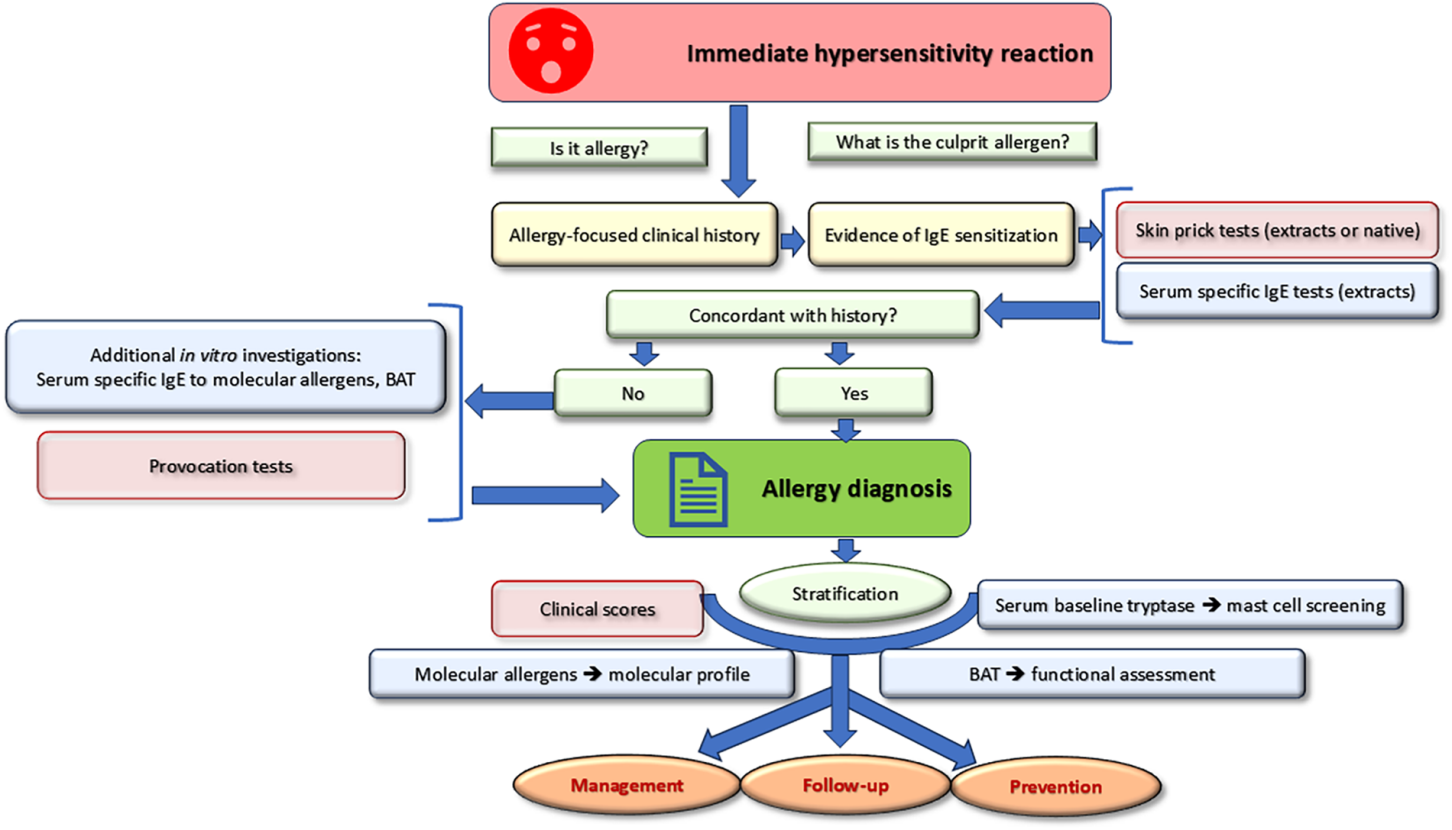
General diagnostic strategy for IgE-mediated immediate hypersensitivity reactions.

Taking a detailed clinical history is the crucial first step to determine the likelihood of an IgE-mediated reaction (Figure 1). The proof of concordant IgE sensitization is the second step allowing to pose the diagnosis of IgE-mediated allergy. Once the diagnosis is confirmed, stratification allows for optimal management. Recent *in vitro* tools such as molecular allergens and basophil activation tests can be used for both diagnostic and stratification purposes. Baseline serum tryptase is a risk marker for the occurrence and severity of systemic hypersensitivity reactions.
